# Intracranial Pressure and Cerebral Perfusion Pressure in Large Spontaneous Intracranial Hemorrhage and Impact of Minimally Invasive Surgery

**DOI:** 10.3389/fneur.2021.729831

**Published:** 2021-08-26

**Authors:** Mais N. Al-Kawaz, Yunke Li, Richard E. Thompson, Radhika Avadhani, Adam de Havenon, Joshua Gruber, Issam Awad, Daniel F. Hanley, Wendy Ziai

**Affiliations:** ^1^Neurosciences Critical Care Division, Department of Neurology, Johns Hopkins University School of Medicine, Baltimore, MD, United States; ^2^Division of Brain Injury Outcomes, Department of Neurology, Johns Hopkins University, Baltimore, MD, United States; ^3^Department of Biostatistics, Johns Hopkins University Bloomberg School of Public Health, Baltimore, MD, United States; ^4^Department of Neurology, Clinical Neurosciences Center, University of Utah, Salt Lake City, UT, United States; ^5^Department of Neurosurgery, University of Chicago Pritzker School of Medicine, Chicago, IL, United States

**Keywords:** intracerebral hemorrage, intracranial pressure, cerebral perfusion pressure, minimally invasive surgeries, intracranial pressure monitoring

## Abstract

**Introduction:** We investigated the effect of hematoma volume reduction with minimally invasive surgery (MIS) on intracranial pressure (ICP) and cerebral perfusion pressure (CPP) in patients with large spontaneous intracerebral hemorrhage (ICH).

**Methods:***Post-hoc* analysis of the Minimally Invasive Surgery Plus Alteplase for Intracerebral Hemorrhage Evacuation (MISTIE III) study, a clinical trial with blinded outcome assessments. The primary outcome was the proportion of ICP readings ≥20 and 30 mmHg, and CPP readings <70 and 60 mm Hg. Secondary outcomes included major disability (modified Rankin scale >3) and mortality at 30 and 365 days. We assessed the relationship between proportion of high ICP and low CPP events and MIS using binomial generalized linear models, and outcomes using multiple logistic regression.

**Results:** Of 499 patients enrolled in MISTIE III, 72 patients had guideline based ICP monitors placed, 34 in the MIS group and 38 in control (no surgery) group. Threshold ICP and CPP events ≥20/ <70 mmHg occurred in 31 (43.1%) and 52 (72.2%) patients respectively. On adjusted analyses, proportion of ICP readings ≥20 and 30 mmHg were significantly lower in the MIS group vs. control group [Odds Ratio (OR) 0.27, 95% Confidence Interval [CI] 0.11–0.63 (*p* = 0.002); OR = 0.18, 0.04–0.75, *p* = 0.02], respectively. Proportion of CPP readings <70 and 60 mm Hg were also significantly lower in MIS patients [OR 0.31, 95% CI 0.15–0.63 (*p* = 0.001); OR 0.30, 95% CI 0.11–0.83 (*p* = 0.02)], respectively. Higher proportions of CPP readings <70 and 60 mm were significantly associated with short term mortality (*p* = 0.04), and (*p* = 0.006), respectively. Long term mortality was significantly associated with higher proportion of time with ICP ≥ 20 (*p* = 0.04), ICP ≥ 30 (*p* = 0.04), and CPP <70 mmHg (*p* = 0.01).

**Conclusion:** Our results are consistent with the hypothesis that surgical reduction of ICH volume decreases proportion of high ICP and low CPP events and that these variables are associated with short- and long-term mortality.

## Introduction

Intracerebral hemorrhage (ICH) represents 10–15% of all strokes worldwide but imposes significant morbidity and mortality. Approximately, 10–30 patients per 100,000 are affected annually with a case fatality as high as 40% at 1 month and 54% at 1 year (1). Deleterious outcomes in ICH are a result of primary and secondary pathologic insults. Primary ICH insult is inflicted mostly by mechanical mass effect secondary to clot formation (2). Further neurologic deterioration in ICH patients can occur due to delayed insult secondary to hematoma growth, intraventricular expansion, and perihematomal edema (2). Hematomal mass effect, evolving perihematomal edema, and perihematomal growth can result in decreased cerebral perfusion pressure (CPP), increased intracranial pressure (ICP), and herniation (2). In a systematic review and meta-analysis, about two-thirds of ICH patients who underwent ICP monitoring demonstrated at least one episode of elevated ICP (3). Despite this common occurrence, little evidence exists to support specific ICP and CPP thresholds in ICH patients and their impact on long term outcomes (4–7). A systematic review and several retrospective studies do suggest, however, that increased ICP level, duration, and variability are associated with worse outcomes and mortality (5, 7–9).

Minimally invasive surgery for ICH can reduce mass effect and may mitigate high ICP and low CPP events. However, post-operative re-hemorrhage and brain edema may oppose this potential benefit. The impact of surgical hematoma reduction on ICP and CPP levels and whether these impact patient outcomes has not been systematically studied.

We hypothesized that patients who had ICP monitors placed who underwent minimally invasive surgery (MIS) using stereotactic aspiration with alteplase would have a lower time burden and incidence of increased ICP and decreased CPP compared to patients with ICP monitors treated with medical management alone.

## Methods

### Design and Study Population

We conducted a *post-hoc* exploratory analysis of data collected in the Minimally Invasive Surgery Plus Alteplase for Intracerebral Hemorrhage Evacuation III (MISTIE III) trial. MSTIE III was a multicenter, randomized, open label, blinded endpoint trial that found image guided, minimally invasive surgery followed by gentle thrombolytic irrigation of the catheterized intracerebral hemorrhage clot decreased mortality, but was neutral on the primary endpoint of improved functional outcome in patients with moderate to large ICH, compared to standard medical management (9). The main inclusion criteria in the trial were (1) age 18 years or older, (2) spontaneous non-traumatic supratentorial ICH with hematoma volume >30 ml and without evidence of an underlying macrovascular cause, (3) presentation within 24 hours of symptom onset, (4) presentation Glasgow Coma Scale (GCS) ≤ 14 or National Institutes of Health Stroke Scale (NIHSS) ≥6, and (5) baseline modified Rankin score (mRS) of <2. Patients randomized to MIS (*n* = 250) received up to nine doses of alteplase every 8 h *via* intrahematomal catheter until hematoma volume was reduced to ≤ 15 ml. The control group received standard medical care (*n* = 249). Details of the methodology and trial results can be found in the primary publication.

In this study, we included all 72 patients who had an ICP monitor placed. ICP monitors were inserted in a guideline supported manner per the neurosurgeons' discretion (10). The trial protocol supported ICP monitoring for “patients with a GCS of 8 or less with two observations over 8 h.” The goals of ICP management were to “sustain ICP below 20 mmHg and to improve the patient's level of consciousness (9).” The protocol specified that placement of an ICP monitor had to be followed by a CT scan of the brain to monitor for ICH stability and any new areas of hemorrhage.

### Standard Protocol Approvals, Registrations, and Patient Consents

The MISTIE III trial was performed at 78 hospitals in the US, Canada, Europe, Australia, and Asia following local institutional review board and country ethics approval. Written informed consent for research was obtained from all participants (or legal representatives or surrogates when applicable). The study was also approved by the Johns Hopkins Hospital institutional review board.

### Measurements and Outcomes

The primary outcomes included occurrence of and percentage of ICP readings ≥20 and 30 mm Hg, and CPP readings <60 and 70 mm Hg. ICP and CPP were recorded every 6 h for up to 6 days after placement of the ICP monitor, including prior to randomization. This time period was intended to include the full duration of the MIS treatment phase. ICP monitors included external ventricular drains (EVDs) and intraparenchymal monitors (IPMs). Choice of monitor placement ipsilateral or contralateral to the ICH was decided by each site's neurosurgical team. ICP and CPP measurements were performed according to standard of care at each center. For EVDs, drainage level and EVD management were directed by site physicians. Validation of q4h measurements with hourly measurements to not miss peak values was previously performed (5). We collected use of any ICP therapy including osmotic therapy, hyperventilation, analgesia, sedation, and where indicated to control ICP, induced coma, but not adherence data to particular thresholds.

Patient demographics and comorbidities were recorded at enrollment. CT scans were evaluated from admission, randomization (termed “stability” when all bleeding had stabilized), and end of treatment (EOT) defined as 24 h after last dose of alteplase or at similar timepoint in the medical group. These were assessed for ICH, IVH, and peri-hematomal edema volumes calculated using semiautomated planimetry, presence of hydrocephalus at diagnosis and EOT, pineal midline shift, and septal midline shift. CT scans were read centrally by trained image readers blinded to treatment and outcomes.

Secondary outcomes were short term and long-term mortality and poor functional outcomes at 30 and 365 days defined by modified Rankin Scale (mRS) score of 4–6. MISTIE III benefited from central blinded adjudication of outcomes using archival video recordings of individual patients.

### Statistical Analysis

Demographics, baseline clinical and radiographic characteristics were compared using Wilcoxon rank sum test for non-normally distributed continuous variables, Student's *t*-test for normally distributed continuous variables, and Pearson Chi Square test for categorical variables. Quantitative data were expressed as median with interquartile range if non-normally distributed, mean with standard deviation for normally distributed data, and as proportions for categorical findings. We tested for intergroup differences for any ICP/CPP threshold event using the chi-squared test for categorical variables, Student's *t*-test for continuous variables, and the Wilcoxon Rank Sum test for ordinal variables. We also graphically compared median daily ICP and CPP levels pre and post-intervention in the MIS group and pre and post-randomization in the medical cohort. Finally, the percentage of ICP readings within each individual subject's record that were above the thresholds of 20 and 30 mmHg (% ICP readings ≥ threshold) and percentage of CPP readings below the thresholds of 60 and 70 mmHg (% CPP readings < threshold) were calculated. Univariable and multivariable analyses of factors associated with % ICP readings above threshold (≥ 20 and ≥30 mmHg) and with % CPP readings below threshold (<70 and <60 mmHg) were performed using binomial generalized linear models, with clustering by patient to adjust for within patient correlations.

Given that there were multiple variables and a limited data set, we used stepwise backward regression including only variables with *P* < 0.05 from the univariable analysis for association with either % ICP or % CPP readings above and below threshold. We also created a second model with four variables which were the most commonly selected in the step-wise regression: age, SBP on admission, IVH (presence vs. absence) and treatment group.

We fit logistic regression models for secondary outcomes to evaluate the contribution of proportion of ICP and CPP threshold events. Due to the small number of patients, models were conservatively adjusted a priori for 5 covariates included in the primary outcome analysis for the MISTIE III trial: age, diagnostic ICH volume, severity of impairment as measured by GCS and clinically established severity variables [IVH and ICH clot location (lobar vs. deep)]. Due to sample size limitations, we were not able to evaluate for effect modification of MIS on proportion of ICP/CPP threshold events. A *p*-value < 0.05 was considered statistically significant. All analyses were carried out in STATA 15 (College Station, TX, USA).

## Results

### Study Population

Of 499 randomized patients in MISTIE III, the cohort included 72 patients (14.4%) who had an ICP monitor placed; 58 patients had EVDs inserted (80.6%), while 14 patients (19.4%) had an IPM. ICP monitors were placed ipsilateral to the hematoma in 6 patients (8.3%). Table 1 compares patients with ICP monitors by treatment group; 34/72 (47.2%) were in the MIS group and 38/72 (52.8%) were in the medical management only group. Supplementary Table 1 compares patients with and without ICP monitors from the MISTIE trial. Patients who had ICP monitors placed were younger, had a lower median admission GCS score and higher NIHSS score, were more likely to require mechanical ventilation, had higher ICH and IVH volume, and had hemorrhages in deep as opposed to superficial (lobar) locations.

**Table 1:** Baseline demographic and radiographic characteristics by treatment group.

**Demographics/Predictors**	**Medical group 38/72 (52.8%)**	**Surgical group 34/72 (47.2%)**	***P*-value**
**Gender**
Female	14 (36.8%)	10 (29.4%)	0.51
Age at consent^*^	56.5 (48–65)	59.5 (48–65)	0.33
**Race**
African American	8 (21.6%)	9 (27.3%)	0.83
Asian	3 (8.1%)	2 (6.1%)	
White	26 (70.3%)	22 (66.7%)	
Hypertension	37 (97.4%)	33 (97.1%)	0.94
Hyperlipidemia	13 (34.2%)	11 (32.4%)	0.87
Prior statin use	3 (7.9%)	1 (2.9%)	0.36
SBP on admission^*^	181.5 (162.5–215)	164 (152–202)	0.13
DBP on admission^*^	98 (85–119)	101 (90–120)	0.71
CAD	7 (18.4%)	5 (14.7%)	0.67
Cocaine use	1 (2.6%)	2 (5.9%)	0.49
Alcohol Abuse	6 (15.8%)	2 (5.9%)	0.18
Anticoagulant use	4 (10.5%)	3 (8.8%)	0.81
Antiplatelet use	11 (29.0%)	8 (23.5%)	0.61
Current Smoker	9 (23.7%)	5 (14.7%)	0.34
Diabetes	7 (18.4%)	8 (23.5%)	0.59
GCS at randomization^*^	8 (7–9)	8 (7–10)	0.84
NIHSS at randomization^*^	23.5 (19–29)	21.5 (17–26)	0.23
ICP therapies used	26 (68.4%)	25 (73.5%)	0.63
EVD inserted	27 (71.1%)	31 (91.2%)	0.03
IPM inserted	11 (28.9%)	3 (8.8%)	
EVD inserted	27 (71.1%)	31 (91.2%)	0.03
EVD ipsilateral to ICH	3 (11.1%)	3 (10.0%)	0.89
Deep ICH location	29 (76.3%)	27 (79.4%)	0.75
Diagnostic septal shift (mm)	4.7 (2.6–6.6)	5.1 (3.5–7.2)	0.39
EOT septal shift (mm)	7.9 (5.7–11.4)	3.9 (2.0–6.2)	<0.001
Delta septal shift (mm)	3.1 (1.4–7.2)	−0.8 (−2.9–1.3)	<0.001
Diagnostic pineal shift (mm)	3.1 (1.7–4.9)	2.1 (1.2–45.0)	0.53
EOT pineal shift	4.0 (2.5–7.1)	2.8 (0–4.4)	0.01
Delta pineal shift (mm)	1.4 (0.1–3.2)	0 (−1.5–1.4)	0.009
IVH present	21 (55.3%)	22 (64.7%)	0.42
Diagnostic IVH volume	0.3 (0–5.4)	4.3 (0.3–10.9)	0.05
Stability IVH volume	2.8 (0–6.9)	5.3 (1.9–9.4)	0.05
EOT IVH volume	1.1 (0.1–5.1)	0.8 (0.3–4.8)	0.83
Diagnostic hydrocephalus	5 (13.5%)	5 (16.1%)	0.76
EOT hydrocephalus	8 (21.1%)	3 (8.8%)	0.15
Diagnostic ICH volume	44.2 (31.9–57.4)	45.4 (32.9–57.9)	0.61
Stability ICH volume	48.5 (38.4–61.7)	48.4 (35.9–69.8)	0.91
EOT ICH volume	47.1 (35.6–66.5)	15.4 (12.2–32.0)	<0.0001
Delta ICH volume	−42.4 (−29.1–21.8)	−3.7 (−0.6–1.8)	<0.0001
EOT <15 mm	0 (0.00%)	16 (47.1%)	0.00
Diagnostic edema volume	26.0 (16.8–33.7)	21.9 (15.4–30.1)	0.52
Stability edema volume	40.5 (36.1–53.0)	31.4 (24.3–42.2)	0.12

*SBP, systolic blood pressure; DBP, diastolic blood pressure; CAD, coronary artery disease; GCS, Glasgow coma scale; NIHSS, NIH stroke scale; EVD, external ventricular drain; IPM, intraparenchymal monitor; ICP, intracranial pressure; ICH, intracerebral hemorrhage; IVH, intraventricular hemorrhage; EOT, end of treatment*.

* *Denotes a value provided in the format of Median (Interquartile Ranger)*.

### Primary Outcomes

**Figure 1:**
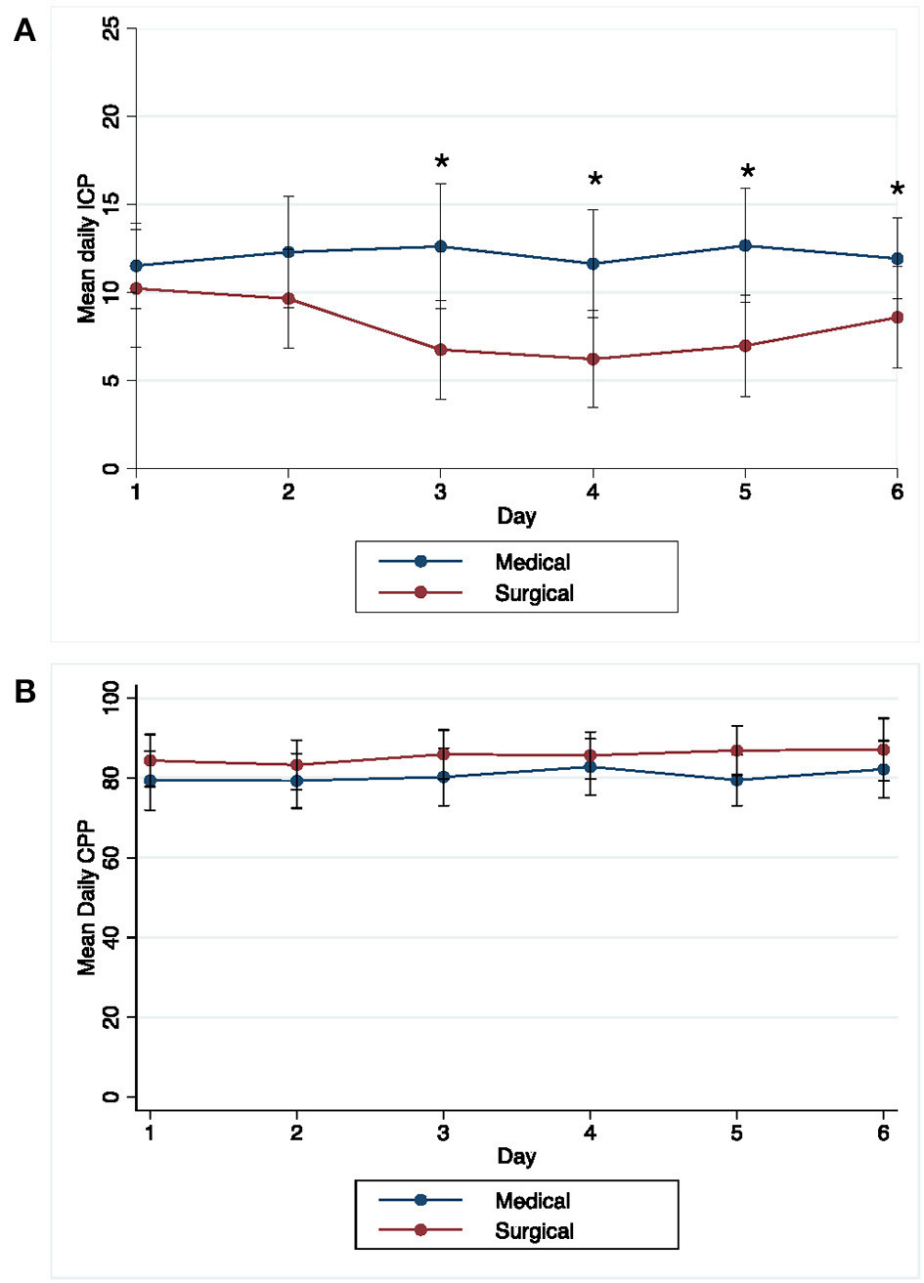
Temporal pattern of daily mean **(**±1 standard deviation) ICP **(A)** and CPP **(B)** over time in MIS and medical groups. *Days with significant difference in mean ICP between medical and surgical groups (*P* < 0.05).

We recorded 1,588 ICP and CPP readings over a median (IQR) of 3 (1–6) days; temporal ICP and CPP trends are shown in Figure 1. The percentage of patients with at least 1 ICP reading above threshold was 43.1 and 16.7% for ≥20 and ≥30 mm Hg, respectively. Supplementary Table 2 compares patients with and without any ICP and CPP threshold event. Any ICP reading ≥20 mm Hg was more likely in younger patients (*p* =0.004), without diabetes (*p* = 0.01), with deep ICH location (*p* = 0.03), higher EOT ICH volume (*p* = 0.04), larger EOT septal shift (*p* = 0.03), and in the medical management arm (*p* = 0.01). Any ICP readings ≥30 mm Hg was more frequent in patients with diabetes (*p* = 0.05). The percentage of patients with at least 1 CPP reading below threshold was 72.2 and 34.7% for below 70 and 60 mmHg, respectively. Any CPP reading <70 mmHg was more likely with presence of IVH (*p* = 0.03). Any CPP reading <60 mm Hg was more likely in patients with less antiplatelet use (*p* = 0.04), hydrocephalus on diagnostic CT (*p* = 0.01) lower GCS (*p* = 0.03) and larger increase in septal shift at end of treatment (*p* = 0.03) (Table 2).

**Table 2:** Baseline demographics and radiographic characteristics for primary outcome measures.

**Demographics/Predictors**	**PC ICP > 20 mm Hg**	**PC ICP > 20 mm Hg**	**PC ICP > 30 mm Hg**	**PC ICP > 30 mm Hg**	**PC CPP <70 mm Hg**	**PC CPP <70 mm Hg**	**PC CPP <60**	**PC CPP <60**
	OR [95% CI]	*P*-value	OR [95% CI]	*P*-value	OR [95% CI]	*P*-value	OR [95% CI]	*P*-value
Male gender	1.89 (0.61–5.86)	0.27	4.30 (0.48–38.5)	0.19	0.81 (0.39–1.66)	0.56	1.11 (0.43–2.93)	0.82
Age at consent	0.93 (0.89–0.97)	0.002	0.88 (0.81–0.96)	0.002	0.99 (0.96–1.02)	0.54	0.98 (0.94–1.03)	0.41
**Race**								
**African American**								
Asian	0.32 (0.03–3.36)	0.34	8.20e-07 (2.89 2e-0.07)	0.98	0.40 (0.05–3.0)	0.38	0.32 (0.03–3.2)	0.33
White	0.36 (0.14–0.92)	0.03	0.13 (0.03–0.53)	0.005	0.73 (0.34–1.56)	0.41	0.39 (0.16–0.95)	0.04
Hypertension	0.46 (0.04–4.96)	0.52	276457.2 (2.89 2e-0.07)	0.99	0.32 (0.07–1.47)	0.14	0.18 (0.04–0.82)	0.03
Hyperlipidemia	0.37 (0.11–1.31)	0.13	0.18 (0.02–2.06)	0.17	0.85 (0.39–1.84)	0.68	0.64 (0.22–1.80)	0.40
Prior statin use	1.09 e-0.06 (2.89 2e-0.07)	0.99	3.89 e-0.6 (2.89 2e-0.07)	0.98	1.44 (0.27–7.55)	0.67	0.48 (0.01–15.93)	0.68
SBP on admission	1.01 (1.00–1.02)	0.12	1.03 (1.01–1.04)	0.004	1.00 (0.99–1.01)	0.57	1.01 (1.00–1.02)	0.04
DBP on admission	1.02 (1.00–1.04)	0.10	1.4 (1.01–1.07)	0.009	1.00 (0.98–1.02)	0.84	1.01 (0.99–1.03)	0.30
CAD	0.62 (0.14–2.75)	0.53	0.32 (0.02–5.72)	0.44	1.20 (0.49–2.96)	0.69	0.91 (0.26–3.18)	0.89
Cocaine use	1.03 (0.12–8.59)	0.98	1.11 (0.05–22.5)	0.95	0.27 (0.01–5.43)	0.39	1.22 e-6 (0-.)	0.98
Alcohol abuse	1.83 (0.57–5.84)	0.31	0.42 (0.02–8.59)	0.57	0.77 (0.23–2.59)	0.68	0.41 (0.05–3.01)	0.38
Anticoagulant use	0.47 (0.04–5.05)	0.53	0.51 (0.02–14.7)	0.70	1.34 (0.42–4.26)	0.62	1.17 (0.25–5.48)	0.84
Antiplatelet use	0.23 (0.04–1.18)	0.08	0.25 (0.03–2.70)	0.25	0.65 (0.27–1.56)	0.34	0.30 (0.07–1.25)	0.10
Current smoker	2.39 (0.93–6.15)	0.07	7.87 (2.09–29.8)	0.002	1.15 (0.49–2.73)	0.75	1.86 (0.71–4.86)	0.21
Diabetes	0.37 (0.97–1.95)	0.24	2.82 2e-0.07 (2.89 2e-0.07)	0.98	1.02 (0.42–2.46)	0.97	0.48 (0.11–2.03)	0.32
GCS at randomization	1.06 (0.86–1.29)	0.59	1.06 (0.79–1.42)	0.71	0.98 (084–1.16)	0.84	0.88 (0.71–1.10)	0.26
NIHSS at randomization	1.04 (0.97–1.11)	0.24	1.12 (1.02–1.23)	0.01	1.02 (0.97–1.07)	0.49	1.04 (0.97–1.11)	0.26
ICP therapies used	1.94 (0.58–6.57)	0.29	15.8 (0.23–1106.2)	0.21	0.85 (0.40–1.81)	0.68	1.46 (0.49–4.30)	0.49
Medical treatment arm	0.22 (0.07–0.69)	0.009	0.09 (0.008–0.96)	0.05	0.30 (0.14–0.65)	0.002	0.30 (0.11–0.82)	0.02
EVD inserted	0.21 (0.08–0.49)	0.00	0.08 (0.02–0.30)	0.00	0.27 (0.14–0.55)	0.00	0.33 (0.13–0.86)	0.03
EVD ipsilateral to ICH	1.02 (0.23–4.45)	0.98	1.42 (0.27–7.58)	0.68	1.28 (0.46–3.55)	0.64	0.98 (0.21–4.63)	0.98
Deep ICH location	3.42 (0.62–19.0)	0.16	9.71 (0.17–543.8)	0.27	1.01 (0.43–2.37)	0.98	1.22 (0.39–3.84)	0.73
Diagnostic septal shift (mm)	0.90 (0.76–1.06)	0.19	0.65 (0.47–0.91)	0.01	0.97 (0.86–1.09)	0.61	0.93 (0.79–1.08)	0.32
EOT septal shift (mm)	1.06 (0.96–1.16)	0.24	1.05 (0.91–1.21)	0.49	1.09 (1.02–1.16)	0.01	1.11 (1.02–1.21)	0.01
Delta septal shift	1.12 (1.01–1.24)	0.03	1.22 (1.04–1.42)	0.01	1.12 (1.04–1.21)	0.002	1.19 (1.08–1.31)	<0.001
Diagnostic pineal shift	1.10 (0.92–1.33)	0.31	1.09 (0.83–1.43)	0.53	1.08 (0.94–1.24)	0.29	1.10 (0.92–1.31)	0.31
EOT pineal shift	1.08 (0.95–1.22)	0.25	1.01 (0.82–1.26)	0.91	1.07 (0.97–1.18)	0.18	1.09 (0.96–1.23)	0.18
Delta pineal shift	1.02 (0.88–1.20)	0.78	0.95 (0.74–1.22)	0.70	1.03 (0.92–1.16)	0.62	1.04 (0.91–1.21)	0.56
Diagnostic hydrocephalus	0.64 (0.15–2.82)	0.56	0.24 (0.01–5.86)	0.38	1.41 (0.60–3.32)	0.44	1.72 (0.62–4.81)	0.31
EOT hydrocephalus	0.58 (0.13–2.59)	0.48	0.40 (0.03–5.14)	0.48	1.18 (0.48–2.87)	0.72	1.39 (0.48–4.06)	0.54
IVH present	0.47 (0.19–1.16)	0.10	0.15 (0.03–0.73)	0.02	0.93 (0.45–1.94)	0.85	0.68 (0.28–1.66)	0.40
Diagnostic IVH volume	0.93 (0.85–1.02)	0.13	0.89 (0.7501.06)	0.18	0.96 (0.91–1.02)	0.18	0.97 (0.91–1.04)	0.38
Stability IVH volume	0.92 (0.83–1.01)	0.09	0.87 (0.72–1.04)	0.13	0.98 (0.93–1.03)	0.34	0.99 (0.93–1.04)	0.63
EOT IVH volume	0.96 (0.86–1.07)	0.43	0.83 (0.62–1.10)	0.20	1.00 (0.95–1.07)	0.75	1.02 (0.94–1.09)	0.70
Diagnostic ICH volume	0.99 (0.97–1.02)	0.49	0.96 (0.92–1.00)	0.04	1.01 (0.99–1.02)	0.84	1.00 (0.97–1.02)	0.64
Stability ICH volume	0.99 (0.96–1.01)	0.33	0.98 (0.94–1.02)	0.34	1.00 (0.99–1.02)	0.60	1.00 (0.98–1.02)	0.91
EOT ICH volume	1.01 (0.99–1.03)	0.22	1.01 (0.99–1.03)	0.42	1.01 (1.00–1.03)	0.02	1.01 (1.00–1.03)	0.16
**Delta ICH volume**								
EOT ICH <15 mm	0.23 (0.04–1.44)	0.12	0.17 (0.008–3.83)	0.27	0.40 (0.13–1.22)	0.11	0.54 (0.15–1.94)	0.34
Diagnostic edema volume	1.02 (1.00–1.06)	0.12	0.98 (0.94–1.04)	0.57	1.02 (0.99–1.05)	0.26	1.00 (0.97–1.04)	0.88
Stability edema volume	1.00 (0.97–1.04)	0.65	1.01 (0.98–1.04)	0.53	1.01 (0.98–1.03)	0.54	1.01 (0.99–1.03)	0.49

*SBP, systolic blood pressure; DBP, diastolic blood pressure; CAD, coronary artery disease; GCS, Glasgow coma scale; NIHSS, NIH stroke scale; EVD, external ventricular drain; IPM, intraparenchymal monitor; IVH, intraventricular hemorrhage; ICH, intracerebral hemorrhage; ICP, intracranial pressure; EOT, end of treatment*.

We used general linear models to assess associations of proportion of ICP and CPP threshold events with MIS, ensuring that the models were not over fitted; first after step-wise backward regression, MIS was significantly associated with decreased proportion of ICP events ≥20 and ≥30 mmHg and with decreased proportion of CPP events <70 and <60 mmHg (Table 3). Second, we controlled for age, presence of IVH, and admission SBP and again found that MIS was associated with decreased threshold events for ICP ≥20, and ≥30, and CPP <70 and <60 mmHg.

**Table 3:** Results of multivariable model for percentage of ICP and CPP threshold readings in MIS (vs. medical management only) group patients.

**Primary outcomes**								
	**Percentage ICP** **>** **20 mm Hg**	**Percentage ICP** **>** **20 mm Hg**	**Percentage ICP** **>** **30 mm Hg**	**Percentage ICP** **>** **30 mm Hg**	**Percentage CPP** **<** **70 mm Hg**	**Percentage CPP** **<** **70 mm Hg**	**Percentage CPP** **<** **60 mm Hg**	**Percentage CPP** **<** **60 mm Hg**
	OR (95% CI)	*P*-value	OR (95% CI)	*P*-value	OR (95% CI)	*P*-value	OR (95% CI)	*P*-value
**MIS group**								
Model 1*	0.27 (0.09–0.83)	0.02	0.04 (0.007–0.21)	<0.001	0.32 (0.14–0.71)	0.005	0.31 (0.11–0.87)	0.03
Model 2^†^	0.27 (0.09–0.83)	0.02	0.08 (0.01–0.59)	0.01	0.31 (0.14–0.71)	0.005	0.32 (0.11–0.92)	0.03

Model 1^*^: Stepwise backward regression model.

*Model 2^†^: Multivariable model adjusted for age, presenting systolic blood pressure, IVH, and treatment group*.

*ICP, intracranial pressure; CPP, cerebral perfusion pressure; MIS, minimally invasive surgery; OR, odds ratio; IVH, intraventricular hemorrhage*.

**Figure 2:**
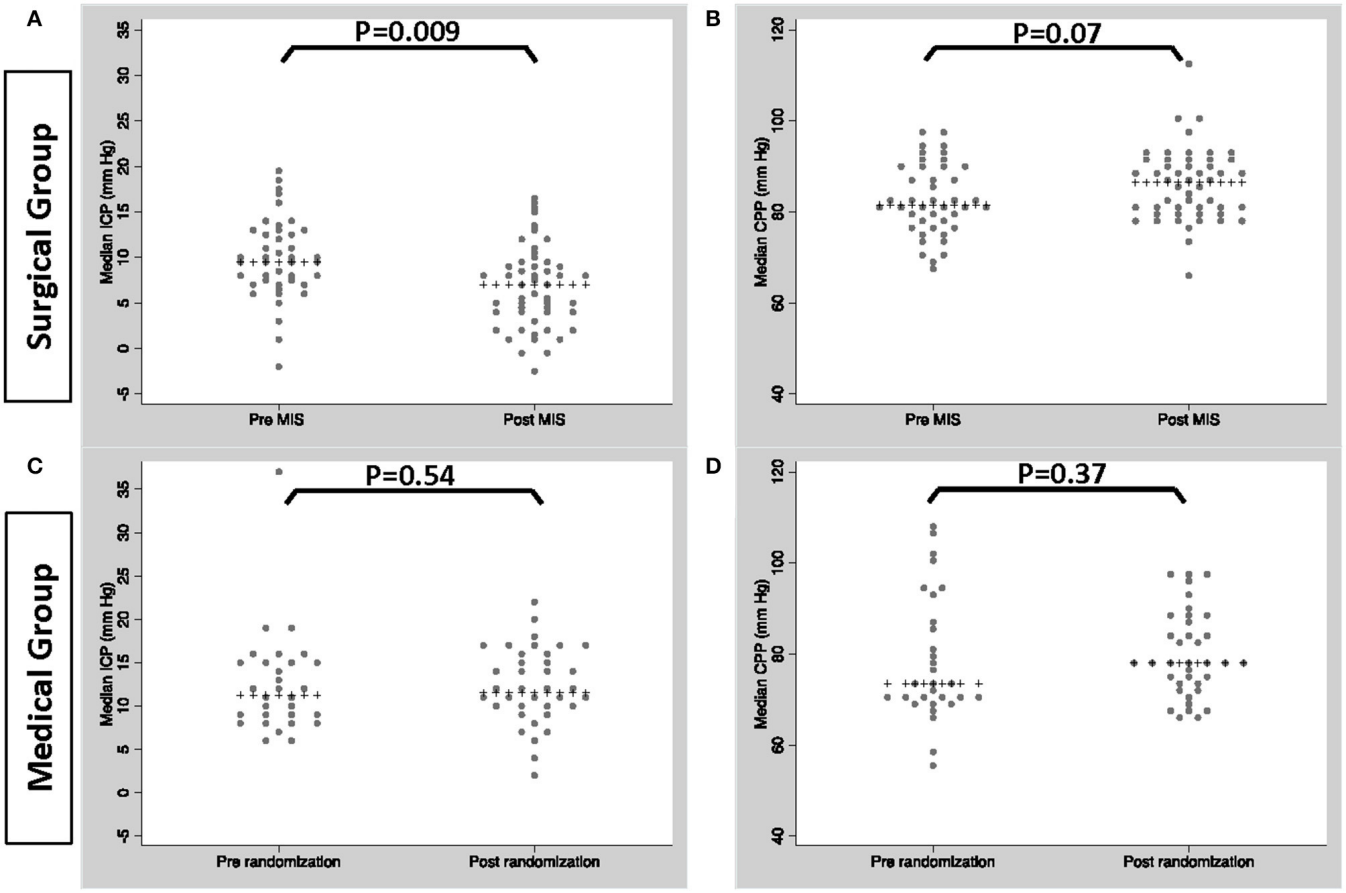
Median ICP/CPP in patients within the MIS group, pre and post MIS **(A,B)** and patients within the medical group pre and post randomization **(C,D)**.

Figure 2 shows median ICP and CPP by time interval. In the MIS group median ICP was significantly lower post MIS compared to pre-MIS (*p* = 0.001); median CPP was higher post MIS, but this difference was not significant (*p* = 0.07). There were no significant differences in median ICP or CPP in the medical group pre and post randomization (correlating to time of surgery).

### Secondary Outcomes

**Figure 3:**
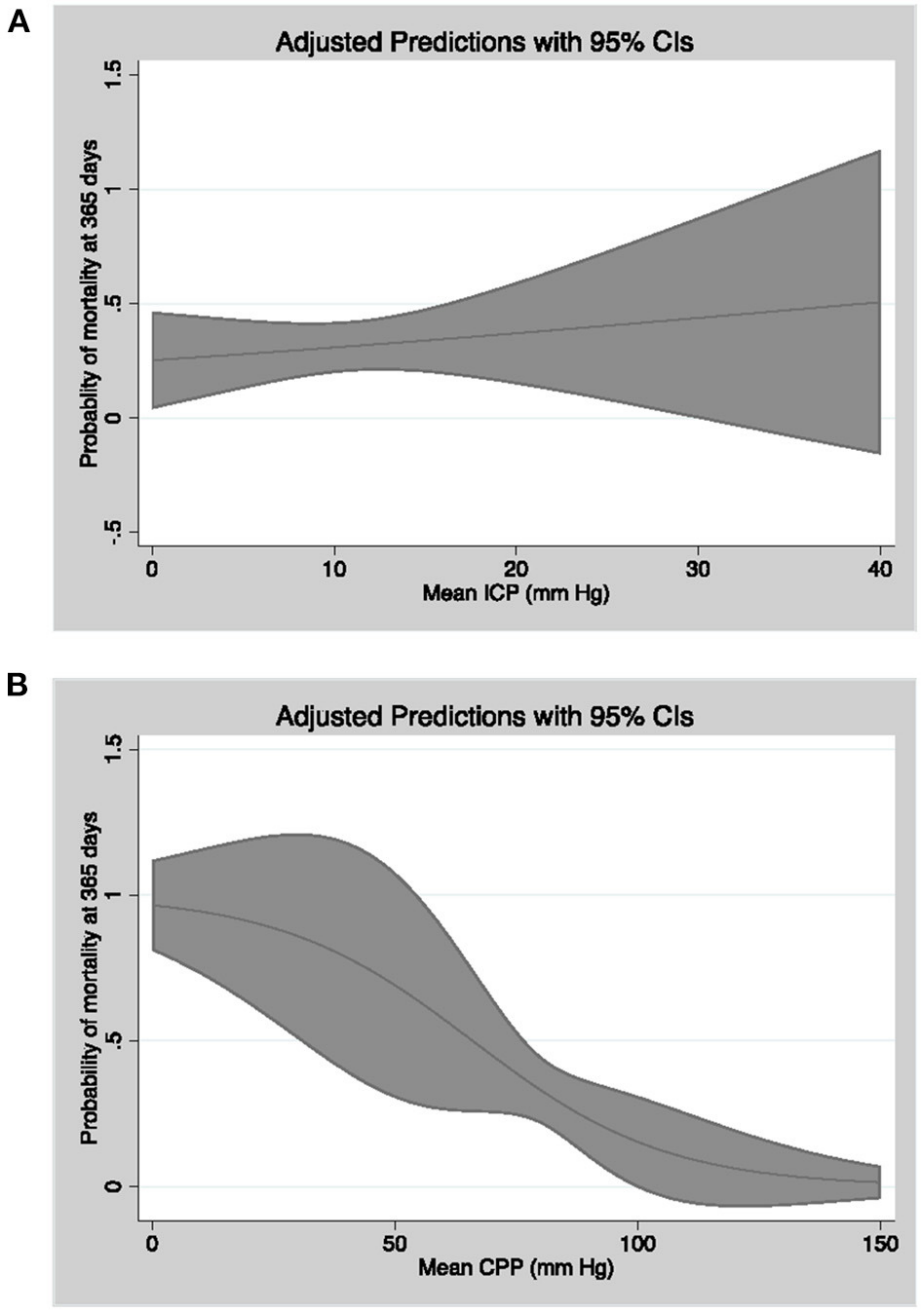
Linear model of probability of mortality at 365 days as a function of mean CPP **(A)** and mean ICP **(B)** with 95% confidence intervals.

In logistic regression models adjusted for the afore mentioned confounders (Table 4), percentage of CPP readings per patient <70 mmHg (OR = 1.71, CI = 1.12–2.59, *p* = 0.01), ICP readings ≥20 mmHg (OR = 1.58, CI = 1.01–2.48, *p* = 0.04), and ICP readings ≥30 mmHg (OR = 1.84, CI = 1.01–3.33, *p* = 0.04) were significantly associated with mortality at 1 year (Figure 3). Percentage of CPP readings <70 and <60 mmHg were also significantly associated with day 30 mortality. Percentage of ICP and CPP readings above/below thresholds were not associated with functional outcomes at 1 year.

**Table 4:** Results of logistic regression models for short and long-term functional outcome and mortality.

**Secondary outcomes at 30 days**	Percentage ICP>20 mm Hg^*^	Percentage ICP>20 mm Hg^*^	Percentage ICP>30 mm Hg^*^	Percentage ICP>30 mm Hg^*^	**Percentage CPP <70 mm Hg**	**Percentage CPP <70 mm Hg**	**Percentage CPP <60 mm Hg**	**Percentage CPP <60 mm Hg**
	OR (95% CI)	*P*-value	OR (95% CI)	*P*-value	OR (95% CI)	*P*-value	OR (95% CI)	*P*-value
Poor neurologic outcome at 30 days	All ICP and CPP thresholds predict poor neurologic function at 30 days perfectly: unable to model							
Mortality at day 30	1.27 (0.78–2.08)	0.34	3.24 (0.61–17.4)	0.28	1.42 (1.02–1.97)	0.04	3.00 (1.36–6.62)	0.006
**Secondary outcomes at 365 days**								
Poor neurologic outcome at 365 days	1.09 (0.69–1.71)	0.72	0.91 (0.62–1.34)	0.64	1.22 (0.86–1.73)	0.26	1.52 (0.58–3.97)	0.39
Mortality at day 365	1.58 (1.01–2.48)	0.04	1.84 (1.01–3.33)	0.04	1.71 (1.12–2.59)	0.01	2.17 (0.88–5.36)	0.09

* *Multivariable model for neurologic function/mortality at 30 and 365 days adjusted for number of ICP/CPP readings, age, GCS, presence of IVH, ICH volume, and deep ICH location*.

*ICP, intracranial pressure; CPP, cerebral perfusion pressure; MIS, minimally invasive surgery; OR, odds ratio; IVH, intraventricular hemorrhage; ICH, intracerebral hemorrhage; GCS, Glasgow coma scale*.

Logistic regression models for short term functional outcomes could not be defined due to all except 1 patient having a poor mRS 4-6 at day 30.

## Discussion

In this secondary analysis of the MISTIE III trial, we found that critical thresholds of ICP ≥20 and 30 and CPP <60 and <70 mmHg are not infrequent in patients with ICP monitors and large ICH. This study is the first to demonstrate lower percentage of monitoring time at high ICP and low CPP values in patients undergoing clot evacuation compared to patients with ICP monitors, but without clot removal. Spending less monitoring time with high ICP and low CPP thresholds was significantly associated with lower mortality at 30 and 365 days. but not with functional outcomes at 1 year.

### Prevalence of ICP and CPP Threshold Events

Elevated intracranial pressure is most likely a common occurrence following moderate to large intracranial hemorrhage where a decision is made to invasively monitor ICP. Godoy et al. reported a pooled prevalence rate of 67% of any episode of intracranial hypertension (ICP >20 mmHg) after ICH in a metanalysis of six studies (3). Factors reported to have a significant association with elevated ICP included GCS at admission, midline shift, age, hemorrhage volume and hydrocephalus. We also found a high rate of occurrence of any ICP ≥20 mmHg which in the medical cohort was 58%, compared to 26% in the surgical cohort. In ICH patients with small parenchymal clots (<30 ml) and large obstructive IVH, this occurrence is even higher at 73% in patients from the CLEAR III trial (11).

### ICP and CPP Time Burden and Outcomes

Optimal ICP and CPP treatment thresholds and associations of threshold events with outcomes in ICH patients are less easily defined (7, 10). Recent evidence points toward time burden, rather than occurrence of any ICP or CPP event, as an important marker associated with outcomes in ICH patients (9). In patients with large IVH causing obstructive hydrocephalus requiring EVD, the percentage of monitoring time with ICP single events from >18 mmHg to >30 mmHg predicted higher short-term mortality, and successive events above 20 mmHg predicted long-term mortality as well. The MISTIE III trial excluded patients with massive IVH, but we found similar associations between long-term mortality and proportion of ICP events above common thresholds of 20 and 30 mmHg for parenchymal ICH volumes >30 ml. Although time at high ICP was not associated with short-term mortality, higher percentage of low CPP readings was significantly associated with higher odds of day 30 mortality both for <70 and <60 mmHg thresholds and at day 365 for <70 mmHg. This again is consistent with data from patients with EVD for large IVH where we previously report CPP as an independent predictor of both short- and long-term mortality and of short-term poor outcome at all thresholds tested from <65 to <90 mm Hg and of long-term poor outcome at <65 and <75 mm Hg. We did not find significant associations between monitored time above and below ICP/CPP thresholds, respectively, with long-term functional outcomes in this study which might be explained by insufficient power due to small sample size, and relatively infrequent ICP events >30 mmHg and CPP events <60 mmHg. Also, it is possible that parenchymal injury from large ICH volume has a greater impact on functional outcomes compared to the analysis of patients in the CLEAR III trial who had relatively small ICH. One study of 243 patients with predominantly supratentorial ICH (median volume 24 ml) showed no correlation between area under the curve of either ICP or CPP and long-term functional outcomes at 12 months, at CPP thresholds of <60 mmHg or <70 mmHg which is consistent with our findings (4).

### Rationale for ICP Monitoring

Despite the high prevalence of increased ICP after ICH, the impact of ICP monitoring on mortality and functional outcomes is not well-established. A secondary analysis of the MISTIE III trial reported that patients with ICP monitors were more likely to have a poor functional outcome at 1 year (77.1 vs. 53.8%) without a significant influence on mortality at 1 year questioning the benefit of ICP monitoring in patients with ICH (11). Patients with ICP monitors, however, had higher clinical severity including higher ICH volume, higher IVH volume and more frequent hydrocephalus on diagnostic CT. Hydrocephalus may be an important clinical factor requiring EVD placement. EVDs in the MIS group were most commonly placed prior to surgery (67.6%) and less commonly at time of MIS. These are association studies, however, and do not imply causality or any treatment recommendations regarding ICP or CPP control. Although ICP was treated aggressively per protocol, ICP and CPP may be markers of outcome but not necessarily “modifiable” therapeutic targets.

### Impact of Minimally Invasive Surgery on ICP and CPP

MISTIE III, one of the largest randomized trials of stereotactic aspiration plus thrombolysis for ICH found a mortality benefit in the surgical cohort, but did not show improvement in functional outcomes at 1 year, with the exception of patients who achieved an end of treatment ICH volume <15 ml (12, 13). The mechanism by which surgical evacuation reduces mortality, and with sufficient clot removal, potentially improves outcomes is likely multifactorial; mitigation of secondary injury pathways, edema formation and both suboptimal ICP and CPP likely play a role. After adjusting for confounding variables, patients who underwent clot volume reduction with minimally invasive surgery experienced a lower monitored time burden of ICP ≥ 20 and 30 mmHg, and CPP <60 and <70 mmHg. The use of mostly EVDs in both medical and surgical patients suggests that CSF drainage likely contributed to ICP control although ICH volume reduction also played a significant role in improving intracranial hemodynamic measures. Sun et al. investigated intraoperative changes in ICP to calculate intraoperative alterations in the “brain-hematoma” pressure gradients (14). Patients undergoing large trauma craniotomy had rapid decreases in ICP and a small “brain-hematoma” pressure gradient after the hematoma was removed. For patients undergoing keyhole endoscopy, ICP decreased slowly and the “brain-hematoma” pressure gradient was initially large, and slowly decreased. The latter procedure more closely resembles stereotactic aspiration with thrombolysis used in MISTIE III and is consistent with our finding of a slow reduction in ICP over several days as hematoma volume was gradually reduced.

## Limitations

These data should be considered alongside a number of limitations. The patient sample size was relatively small, and may not be generalizable to all large ICH volume patients, given the stringent inclusion criteria of a clinical trial. We attempted to prevent over-fitting of multivariable models and may have missed important confounders. Most importantly is confounding by indication since ICP monitors were placed at the discretion of the treating physician, and most commonly in higher severity patients. However, the randomized design mitigates this concern with respect to the MIS intervention. We included patients with both intraparenchymal monitors and patients with EVDs which introduces bias into ICP readings, and the effect of CSF drainage. However, a similar number of patients in the medical and surgical treatment groups had ICP monitors placed. Despite these limitations, this dataset was systematically monitored for correctness, has well-defined objective inclusion criteria, blinded assessment of outcome and adds important information about the characteristics and sequelae of elevated ICP and low CPP in patients with ICH treated with surgical evacuation. While the population studied is small, it is important to note that a robust relationship between mitigation of ICP/CPP thresholds and mortality was identified, similar to that of another large trial for IVH (15).

## Conclusion

This study supports the concept that ICH volume reduction with minimally invasive surgery decreases monitored time spent with high ICP and low CPP in patients with large ICH. Decreasing high ICP and low CPP burden is associated with improved short- and long-term mortality and may represent a mechanism by which mortality outcomes are improved by minimally invasive surgery.

## Data Availability Statement

The original contributions presented in the study are included in the article/Supplementary Material, further inquiries can be directed to the corresponding author/s.

## Ethics Statement

The studies involving human participants were reviewed and approved by Johns Hopkins Hospital institutional review board. The patients/participants provided their written informed consent to participate in this study.

## Author Contributions

MA-K and WZ had full access to all of the data in the study and take responsibility for the integrity of the data and the accuracy of the data analysis. WZ and MA-K: study concept and design. MA-K, DH, and WZ: acquisition, analysis, or interpretation of data. MA-K and WZ: drafting of the manuscript. MA-K, YL, RT, RA, AH, JG, IA, DH, and WZ: critical revision of the manuscript for important intellectual content. MA-K, WZ, and RT: statistical analysis. WZ and DH: administrative, technical, or material support. WZ: study supervision. All authors contributed to the article and approved the submitted version.

## Conflict of Interest

IA and DH were awarded significant research support for Minimally Invasive Surgery Plus Alteplase for Intracerebral Hemorrhage Evacuation (MISTIE) III by NIH/NINDS grant U01NS080824. WZ is supported by grants R01NS102583, R01AG06993, U01NS106513 and U01NS080824. AH reports investigator-initiated funding from Regeneron, Amgen, and AMAG pharmaceuticals. IA reports grants from NIH outside the submitted work. DH reports grants from NIH including BEACH (NIA): R01AG06993, TIC (NCATS): U24TR001609, and personal fees from BrainScope, Neurotrope, Op2Lysis, and Portola Pharmaceuticals, outside the submitted work. WZ is an associate editor for Neurocritical Care and an assistant editor for Stroke and has received data monitoring committee fees from C.R. Bard, Inc. outside the submitted work. The remaining authors declare that the research was conducted in the absence of any commercial or financial relationships that could be construed as a potential conflict of interest.

## Publisher's Note

All claims expressed in this article are solely those of the authors and do not necessarily represent those of their affiliated organizations, or those of the publisher, the editors and the reviewers. Any product that may be evaluated in this article, or claim that may be made by its manufacturer, is not guaranteed or endorsed by the publisher.
