# Supplementary material for: Intracranial Pressure and Cerebral Perfusion Pressure in Large Spontaneous Intracranial Hemorrhage and Impact of Minimally Invasive Surgery
Source: Front Neurol. 2021 Aug 26;12:729831. doi: 10.3389/fneur.2021.729831 (PMC8427275; doi:10.3389/fneur.2021.729831)
Supplement: Supplementary file 1 [file Table_1.DOCX]

**Supplemental Table 1:** Characteristics of ICP monitored and non-monitored patients in the MISTIE III cohort

| **Demographics/Predictors** | **ICP monitored 72/499 (14.4%)** | **No ICP monitoring 427/499 (85.6%)** | **p-value** |
| --- | --- | --- | --- |
| Gender  Female | 24 (33.3%) | 170 (39.8%) | 0.30 |
| Age at consent* | 58 (48.5-64) | 63 (53-71) | 0.004 |
| Race  African American  Asian  White | 17 (23.6%)  5 (6.9%)  49 (68.1%) | 70 (16.4%)  25 (5.9%)  325 (76.1%) | 0.14 |
| Hypertension | 70 (97.2%) | 411 (96.3%) | 0.68 |
| Hyperlipidemia | 24 (33.3%) | 165 (38.6%) | 0.39 |
| CAD | 12 (16.7%) | 60 (14.1%) | 0.56 |
| Cocaine use | 3 (4.2%) | 17 (4.0%) | 0.94 |
| Alcohol Abuse | 8 (11.1%) | 60 (14.1%) | 0.51 |
| Anticoagulant use | 7 (9.7%) | 27 (6.3%) | 0.29 |
| Antiplatelet use | 19 (26.4%) | 125 (29.3%) | 0.62 |
| Current Smoker | 17 (19.4%) | 75 (17.6%) | 0.70 |
| Diabetes | 15 (20.8%) | 124 (29.1%) | 0.15 |
| GCS at randomization* | 8 (7-10) | 11 (9-13) | <0.001 |
| NIHSS at randomization* | 23 (18-27) | 19 (15-22) | <0.001 |
| Deep ICH location | 56 (77.8%) | 251 (58.8%) | 0.002 |
| Diagnostic hydrocephalus | 10 (14.7%) | 31 (7.5%) | 0.05 |
| Diagnostic IVH volume | 2.6 (0-8.3) | 0 (0-1.1) | <0.001 |
| Stability IVH volume | 3.95 (0.4-8.5) | 0.2 (0-2.2) | <0.001 |
| EOT IVH volume | 1.1 (0.2-5.0) | 0.2 (0-1.4) | <0.001 |
| Diagnostic ICH volume | 44.5 (31.2-56.3) | 41.4 (30.8-54.5) | 0.37 |
| Stability ICH volume | 48.5 (37.7-65.9) | 45.3 (35.3-57.5) | 0.07 |
| EOT ICH volume | 36.1 (15.5-49.1) | 28.0 (11.6-43.9) | 0.01 |
| EOT less than 15mm | 16 (22.2%) | 132 (31.3%) | 0.12 |

Abbreviations- CAD: Coronary artery disease; GCS: Glasgow Coma scale; NIHSS: NIH stroke scale; IVH: intraventricular hemorrhage; ICH: intracerebral hemorrhage; ICP: Intracranial pressure; EOT: End of treatment.

**Supplemental Table 2:** Demographics and radiographic characteristics of patients with any incident of ICP ≥20 and 30 mmHg and any incident of CPP <70 and 60 mmHg.

| **Demographics/Predictors** | **Any incident of ICP**≥**20 mmHg**  **31/72 (43.1%)** | **No incident of ICP**≥**20 mmHg**  **41/72 (56.9%)** | **p-value** | **Any incident of ICP**≥**30 mmHg**  **12/72 (16.7%)** | **No incident of ICP**≥**30 mmHg**  **60/72 (83.3%)** | **p-value** | **Any incident of CPP <70 mmHg**  **52/72 (72.2%)** | **No incident of CPP<70**  **20/72 (27.8%)** | **p-value** | **Any incident of CPP <60 mmHg**  **25/72 (34.7%)** | **No incident of CPP<60**  **47/72 (65.3%)** | **p-value** |
| --- | --- | --- | --- | --- | --- | --- | --- | --- | --- | --- | --- | --- |
| Gender  Female | 8 (33.3%) | 21 (66.7%) | 0.24 | 3 (25.0%) | 21 (35.0%) | 0.51 | 18 (75.0%) | 6 (25.0%) | 0.71 | 10 (41.7%) | 14 (58.3%) | 0.38 |
| Age at consent* | 54 (47-59) | 62 (52-66) | 0.004 | 55.5 (42-61) | 59 (49.5-65) | 0.11 | 58.5 (52-64) | 53.5 (45.5-64) | 0.46 | 59 (53-67) | 57 (48-64) | 0.29 |
| Race  African American  Asian  White | 10 (32.3%)  3 (9.7%)  18 (58.1%) | 7 (17.2%)  2 (5.0%)  31 (77.5%) | 0.21 | 5 (41.7%)  0 (0.0%)  7 (58.3%) | 12 (20.3%)  5 (8.5%)  42 (71.2%) | 0.21 | 12 (23.1%)  3 (5.8%)  37 (71.2%) | 5 (26.3%)  2 (10.5%)  12 (63.2%) | 0.73 | 5 (20.0%)  2 (8.0%)  18 (72.0%) | 12 (26.1%)  3 (6.5%)  31 (67.4%) | 0.84 |
| Hypertension | 30 (96.8%) | 40 (97.6%) | 0.84 | 12 (100.0%) | 58 (96.7%) | 0.52 | 51 (98.1%) | 19 (95.0%) | 0.48 | 24 (96.0%) | 46 (97.9%) | 0.65 |
| Hyperlipidemia | 7 (22.6%) | 17 (41.5%) | 0.09 | 3 (25.0%) | 21 (35.0%) | 0.51 | 15 (28.9%) | 9 (45.0%) | 0.19 | 10 (40.0%) | 14 (29.8%) | 0.38 |
| Prior statin use | 0 (0.0%) | 4 (9.8%) | 0.07 | 0 (0.0%) | 6 (6.7%) | 0.36 | 2 (3.9%) | 2 (10.0%) | 0.31 | 1 (4.0%) | 3 (6.4%) | 0.67 |
| SBP on admission* | 179 (159-219) | 174 (158.5-206.5)* | 0.67 | 172.5 (156-203) | 210 (179-222) | 0.05 | 175 (158-210) | 180 (163-228) | 0.51 | 174 (159-220) | 177 (156-210) | 0.95 |
| DBP on admission* | 102 (96-119) | 98 (82-120) | 0.28 | 119 (100-127) | 98 (85-114) | 0.07 | 98.5 (85-119) | 103.5 (92-125) | 0.35 | 98 (85-108) | 102 (90-123) | 0.36 |
| CAD | 5 (16.1%) | 7 (17.1%) | 0.92 | 2 (16.7%) | 10 (16.7%) | 1.00 | 7 (13.5%) | 5 (25.0%) | 0.24 | 5 (20.0%) | 7 (14.9%) | 0.58 |
| Cocaine use | 2 (6.5%) | 1 (2.4%) | 0.40 | 1 (8.3%) | 2 (3.3%) | 0.43 | 3 (5.8%) | 0 (0.0%) | 0.27 | 0 (0.0%) | 3 (6.4%) | 0.20 |
| Alcohol Abuse | 5 (16.1%) | 3 (7.3%) | 0.24 | 2 (16.7%) | 6 (10.0%) | 0.51 | 5 (9.6%) | 3 (15.0%) | 0.52 | 2 (8.0%) | 6 (12.8%) | 0.54 |
| Anticoagulant use | 2 (28.6%) | 5 (12.2%) | 0.42 | 1 (8.3%) | 6 (10.0%) | 0.86 | 4 (7.7%) | 3 (15.0%) | 0.35 | 2 (8.0%) | 5 (10.6%) | 0.72 |
| Antiplatelet use | 6 (19.4%) | 13 (31.7%) | 0.24 | 2 (16.7%) | 17 (28.3%) | 0.41 | 12 (23.1%) | 7 (35.0%) | 0.31 | 3 (12.0%) | 16 (34.1%) | 0.04 |
| Current Smoker | 8 (25.8%) | 6 (14.6%) | 0.24 | 3 (25.0%) | 11 (18.3%) | 0.59 | 9 (17.3%) | 5 (25.0%) | 0.46 | 5 (20.0%) | 9 (19.2%) | 0.93 |
| Diabetes | 2 (6.5%) | 13 (31.7%) | 0.009 | 0 (0.0%) | 15 (25.0%) | 0.05 | 10 (19.2%) | 5 (25.0%) | 0.60 | 4 (16.0%) | 11 (23.4%) | 0.46 |
| GCS at randomization* | 8 (7-10) | 9 (7-10) | 0.74 | 8 (7-10) | 8 (7-10) | 0.99 | 8 (7-9) | 9 (7.5-11) | 0.12 | 7 (7-9) | 9 (7-10) | 0.03 |
| NIHSS at randomization* | 23 (18-29) | 22 (18-26) | 0.72 | 25 (23-30.5) | 22 (18-26.5) | 0.12 | 24 (18.5-29.5) | 20 (17.5-25.5) | 0.11 | 24 (22-30) | 21 (18-26) | 0.11 |
| ICP therapies used | 25 (80.7%) | 26 (63.4%) | 0.11 | 11 (91.7%) | 40 (66.7%) | 0.08 | 36 (69.2%) | 15 (75.0%) | 0.63 | 19 (76.0%) | 32 (68.1%) | 0.48 |
| Medical treatment arm | 22 (71.0%) | 16 (39.1%) | 0.007 | 9 (75.0%) | 29 (48.3%) | 0.09 | 21 (40.4%) | 13 (65.0%) | 0.06 | 17 (68.0%) | 21 (44.7%) | 0.06 |
| EVD inserted  Parenchymal Monitors | 24 (77.4%)  7 (22.6%) | 34 (82.9%)  7 (17.1%) | 0.56 | 10 (83.3%)  2 (16.7%) | 48 (80.0%)  12 (20.0%) | 0.79 | 43 (82.7%)  9 (17.3%) | 15 (75.0%)  5 (25.0%) | 0.46 | 21 (84.0%)  4 (16.0%) | 37 (78.7%)  10 (21.3%) | 0.59 |
| EVD ipsilateral to ICH | 3 (12.5%) | 3 (9.1%) | 0.68 | 1 (10.0%) | 5 (10.6%) | 0.95 | 5 (11.6%) | 1 (7.1%) | 0.64 | 1 (4.8%) | 5 (13.9%) | 0.28 |
| Deep ICH location | 28 (68.3%) | 28 (90.3%) | 0.03 | 11 (91.7%) | 45 (75.0%) | 0.21 | 12 (23.1%) | 4 (20.0%) | 0.78 | 5 (20%) | 11 (23.1%) | 0.74 |
| Diagnostic septal shift (mm) | 5.3 (2.7-7.6) | 4.9 (3.0-6.6) | 0.72 | 3.5 (1.9-6.6) | 5.2 (3.0-7.6) | 0.24 | 5.3 (2.9-7.6) | 3.8 (2.3-5.8) | 0.12 | 5.3 (2.7-7.2) | 4.9 (3.0-6.7) | 0.99 |
| EOT septal shift (mm) | 7.9 (5.0-11.1) | 5.3 (2.6-7.3) | 0.03 | 7.1 (4.7-9.7) | 5.9 (3.1-9.8) | 0.33 | 6.2 (3.2-10.9) | 4.9 (2.1-7.5) | 0.27 | 8.0 (4.0-11.8) | 5.6 (2.8-7.6) | 0.04 |
| Delta septal shift | 1.4 (-0.4-6.4) | 0 (-2.1- 3.1) | 0.15 | 1.6 (0.96- 7.7) | 1.3 (-1.7 – 3.7) | 0.15 | 1.3 (-0.94 – 5.2) | 1.6 (-1.7-3.7) | 0.87 | 2.3 (-0.26-7.2) | 0.6 (-1.8-3.1) | 0.03 |
| Diagnostic pineal shift (mm) | 3 (1.7-5.2) | 2.3 (0-4.9) | 0.23 | 2.5 (1.1-4.1) | 2.8 (1.2-5.0) | 0.68 | 3.0 (1.2-5.1) | 2.3 (1.1-4.5) | 0.49 | 3.0 (1.2-5.3) | 2.3 (1.5-4.8) | 0.59 |
| EOT pineal shift (mm) | 3.7 (2.4-6.9) | 2.8 (1.9-5.5) | 0.11 | 3.6 (2.1-4.2) | 3.5 (2.1-6.1) | 0.99 | 3.7 (2.2-5.5) | 3.1 (1.4-7.3) | 0.83 | 4.4 (2.4-6.5) | 3.1 (2.0-4.3) | 0.17 |
| Delta pineal shift | 1.3 (-0.4- 2.7) | 0.60 (-1.1-2.4) | 0.47 | 0.7(-0.9-2.8) | 0.8 (-0.9-2.7) | 0.99 | 0.8 (-1.1-2.4) | 0.6 (0-3.1) | 0.48 | 0.87 (-0.4-2.8) | 0.6 (-1.3-2.5) | 0.52 |
| Diagnostic hydrocephalus | 6 (20.7%) | 4 (10.3%) | 0.23 | 2 (16.7%) | 8 (14.3%) | 0.83 | 9 (18.0%) | 1 (5.6%) | 0.21 | 7 (29.2%) | 3 (6.8%) | 0.01 |
| EOT hydrocephalus | 6 (19.4%) | 5 (12.2%) | 0.41 | 2 (16.7%) | 9 (15.0%) | 0.88 | 8 (15.4%) | 3 (15.0%) | 0.97 | 6 (24.0%) | 5 (10.6%) | 0.13 |
| IVH present | 18 (58.1%) | 25 (61.0%) | 0.81 | 7 (58.3%) | 36 (60.0%) | 0.91 | 35 (67.3%) | 8 (40.0%) | 0.03 | 15 (60.0%) | 28 (59.6%) | 0.97 |
| Diagnostic IVH volume | 1.7 (0-10.1) | 3.1 (0-7.4) | 0.96 | 1 (0-13.9) | 3.1 (0-7.6) | 0.85 | 3.3 (0-9.9) | 0 (0-5.9) | 0.06 | 2.1 (0-9.9) | 3 (0-8) | 0.98 |
| Stability IVH volume | 3 (0.1-9.7) | 4.5 (1.8-7.2) | 0.55 | 1.3 (0-13) | 4.3 (0.5-7.1) | 0.65 | 4.6 (0.6-10.5) | 2.6 (0.3-5.2) | 0.17 | 4.5 (0.9-11.3) | 3.8 (0.2-7) | 0.59 |
| EOT IVH volume | 1.1 (0.1-5.8) | 1 (0.2-4.2) | 0.85 | 0.4 (0-3.1) | 1.5 (0.2-5.3) | 0.25 | 1.6 (0.2-6.3) | 0.6 (0.2-2.2) | 0.32 | 1.1 (0.3-9.6) | 1 (0.1-4.8) | 0.48 |
| Diagnostic ICH volume | 44.1 (33.1-50.8) | 45.4 (31.2-68.6) | 0.60 | 35.6 (27.4-50.2) | 45.3 (31.9-57.9) | 0.30 | 45.7 (33.1-57.4) | 36.2 (26.7-46.5) | 0.08 | 45.6 (27.7-51.7) | 44.1 (31.6-57.1) | 0.81 |
| Stability ICH volume | 46.6 (38.9-58.3) | 51.3 (35.7-69.6) | 0.54 | 42.3 (38.2-55.8) | 49.8 (36.9-66.5) | 0.41 | 48.5 (39.1-68.0) | 45.3 (34.1-61.7) | 0.39 | 50.7 (41.2-69.6) | 47.0 (35.6-65.5) | 0.26 |
| EOT ICH volume | 43.6 (29.0-52.0) | 31.4 (14.0-41.7) | 0.04 | 40.4 (27.6-50.0) | 35.7 (15.4-49.1) | 0.58 | 36.9 (19.9-49.1) | 24.9 (12.1-50.4) | 0.25 | 40.6 (31.4-53.3) | 31.9 (13.9-47.5) | 0.18 |
| EOT less than 15mm | 4 (12.9%) | 12 (29.3%) | 0.10 | 2 (16.7%) | 14 (23.3%) | 0.61 | 9 (17.3%) | 7 (35.0%) | 0.11 | 4 (16.0%) | 12 (25.5%) | 0.35 |
| Diagnostic edema volume | 23.7 (19.2-30.7) | 21.8 (15.4-33.7) | 0.61 | 21.1 (20.8-23.7) | 22.2 (15.8-32.4) | 0.83 | 22.1 (18.1-29.4) | 22.1 (11-32.4) | 0.59 | 22.2 (20.1-27.9) | 21.8 (16.8-30.7) | 0.73 |
| Stability edema volume | 38.7 (31.4-43.9) | 31.6 (24.3-42.2) | 0.31 | 31.4 (24.3-43.5) | 37.1 (28.4-43.3) | 0.94 | 38.3 (30.2-43.9) | 32.1 (24.3-40.7) | 0.21 | 41.5 (31.4-63.8) | 32.3 (24.3-40.7) | 0.06 |

Abbreviations- SBP: Systolic blood pressure; DBP: Diastolic blood pressure; CAD: Coronary artery disease; GCS: Glasgow Coma scale; NIHSS: NIH stroke scale; EVD External ventricular drain; IPM: intraparenchymal monitor; IVH: intraventricular hemorrhage; ICH: intracerebral hemorrhage; ICP: Intracranial pressure; EOT: End of treatment.
